# Cryptotanshinone has curative dual anti-proliferative and immunotherapeutic effects on mouse Lewis lung carcinoma

**DOI:** 10.1007/s00262-019-02326-8

**Published:** 2019-04-10

**Authors:** Shuo Liu, Zhen Han, Anna L. Trivett, Hongsheng Lin, Sean Hannifin, De Yang, Joost J. Oppenheim

**Affiliations:** 1 Cancer and Inflammation Program, Center for Cancer Research, National Cancer Institute, Frederick National Laboratory for Cancer Research (FNLCR), Rm 21-89/31-19, Bldg 560, 1050 Boyles Street, Frederick, MD 21702-1201 USA; 2 Guang An Men Hospital of China Academy of Chinese Medical Sciences, Beijing, China

**Keywords:** Cryptotanshinone, Lewis lung carcinoma, Dendritic cells, Cancer therapeutic, Cell-cycle arrest

## Abstract

Lung cancer is currently the leading cause of cancer-related mortality with very limited effective therapy. Screening of a variety of traditional Chinese medicines (TCMs) for their capacity to inhibit the proliferation of human lung cancer A549 cells and to induce the in vitro maturation of human DCs led to the identification of cryptotanshinone (CT), a compound purified from the TCM *Salvia miltiorrhiza Bunge*. Here, CT was shown to inhibit the proliferation of mouse Lewis lung carcinoma (LLC) cells by upregulating p53, downregulating cyclin B1 and Cdc2, and, consequently, inducing G2/M cell-cycle arrest of LLC cells. In addition, CT promoted maturation of mouse and human DCs with upregulation of costimulatory and MHC molecules and stimulated DCs to produce TNFα, IL-1β, and IL-12p70, but not IL-10 in vitro. CT-induced maturation of DCs depended on MyD88 and also involved the activation of NF-κB, p38, and JNK. CT was effective in the treatment of LLC tumors and, when used in combination with low doses of anti-PD-L1, cured LLC-bearing mice with the induction of subsequent anti-LLC long-term specific immunity. CT treatment promoted T-cell infiltration and elevated the expression of genes typical of Th1 polarization in LLC tumor tissue. The therapeutic effect of CT and low doses of anti-PD-L1 was reduced by depletion of CD4 and CD8 T cells. This paper provides the first report that CT induces immunological antitumor activities and may provide a new promising antitumor immunotherapeutic.

**Electronic supplementary material:**

The online version of this article (10.1007/s00262-019-02326-8) contains supplementary material, which is available to authorized users.

## Introduction

Lung cancer is the leading cause of cancer-related mortality worldwide, with a 5-year overall survival rate of only 15% for all stages of patients [1]. The majority (~ 75%) of lung cancer patients are diagnosed at an advanced stage of the disease [1–3]. Nonspecific cytotoxic chemotherapy is associated with severe side effects, while surgery is not effective for late-stage disease [3]. Targeted therapy against epidermal growth factor (e.g., erlotinib, afatinib, etc.) or anaplastic lymphoma kinase (crizotinib and ceritinib) is helpful only for a small subgroup of patients with relevant targetable genomic alterations [2]. Recently, checkpoint inhibitor antibodies against PD1 (Nivolumab and Pembrolizumab) or PD-L1 (Duvalumab, Atezolizumab, and Avelumab) have been used to treat lung cancer patients with an overall response rate of approximately 20–25% [3]. Therefore, the development of additional effective therapies for lung cancers is still needed.

Vertebrates generate specific immune responses against malignant tumor cells such as the production of IFNγ and tumor-specific cytotoxic CD8 T cells [4, 5]. At advanced stages of cancers, including lung cancer, the tumor tissues become highly immunosuppressive due to the infiltration by immunoinhibitory cells such as Tregs and MDSCs and/or the generation of inhibitory factors such as PD-1/PD-L1, lymphocyte activation gene-3, IL-10, and TGFβ [5–8]. The immunosuppressive tumor microenvironment yields at least two dire immunological consequences. One is to nullify the cancer cell-killing capacity of preexisting CTLs, which can be countered by checkpoint inhibitor antibodies [9]. The other is to incapacitate antigen-presenting DCs in the tumor tissue by preventing DC maturation and presentation of tumor-associated antigens to naïve T cells in the secondary lymphoid organs, thus, hampering the generation of additional tumor-specific CTLs [10–12].

Increased cancer cell number and tumor size play an essential role in cancer immunosuppression by producing many immunosuppressive factors such as TGFβ [8, 13], which then promotes IL-10 production and generation of Tregs and MDSCs [8, 13–15]. Therefore, reducing tumor burden would facilitate curtailing the immunosuppressive microenvironment in lung cancer. Another means of overcoming the immunosuppressive microenvironment would be to directly activate DCs in lung cancer tissues, so that they regain their antigen-presenting capacity and produce proinflammatory cytokines (such as TNFα and IL-12) capable of countering the immunosuppressive cytokines. Thus, any agent capable of simultaneously inhibiting the proliferation of lung cancer cells and inducing the maturation of DCs would be highly desirable.

TCMs are widely employed in China and some southeastern Asian countries for the treatment of cancers. The major theoretical principle for treating cancers in TCM is ‘Gu Ben Qing Yuan’, which in English grossly means ‘simultaneously reinforcing the body’s protective anticancer immune response and eliminating cancer cells. We speculated that certain TCMs might possess these dual capacities. We, therefore, screened more than a dozen of TCMs and purified TCM compounds that are used for the treatment of cancer patients. This led to the identification of cryptotanshinone (CT), a compound isolated from the TCM Danshen/*Salvia miltiorrhiza Bunge* [16], as an inhibitor of the proliferation of a human lung cancer cell line A549 and concomitant inducer of the maturation of human DCs. CT is biochemically well characterized [17], can be chemically synthesized [18], and is available commercially with more than 98% purity. We investigated the mechanistic basis by which CT inhibited the proliferation of lung cancer cells and stimulated the maturation of DCs. Furthermore, the capacity of CT to treat lung cancer was evaluated using the mouse Lewis lung carcinoma model. The data demonstrate that CT inhibited the proliferation of lung cancer cells, induced DC maturation via distinct signaling pathways, and had a curative effect on Lewis lung carcinoma in immunocompetent mice.

## Materials and methods

### Reagents

Compounds purified from various TCMs, including CT, were obtained from the National Institutes for Food and Drug Control (Beijing, China). CT was dissolved at a stock concentration of 15 ~ 20 mg/ml in DMSO and diluted into physiologic solutions or medium for experiments. LPS (*E. coli* O55:B5) was from Sigma. Anti-PD-L1 (clone 10F.9G2) and control rat IgG2b (clone LTF-2) were purchased from BioXCell (West Labanon, NH).

### Proliferation assay

A549 or LLC cells were seeded into a 96-well flat-bottomed tissue culture plate at 4 × 10^3^/well in appropriate medium and cultured in a CO_2_ incubator (37 °C humidified air-containing 5% CO_2_) overnight. After adding CT, the cells were incubated for 48 h and pulsed with tritiated thymidine (^3^H-TdR, New England Nuclear, North Billerica, MA) at 0.5 µCi/well for the last 4 h. At the end of culture, the cells were collected onto a membrane with a 96-well harvester (INOTECH_AG_ IH-280, Dottikon, Switzerland) to measure ^3^H-TdR incorporation (CPM) using an automatic MicroBeta counter (Wallac). The change in the percentage (%) of cell proliferation was calculated as: % Proliferation = (CPM with compound − CPM blank)/(CPM without compound − CPM blank) × 100. The concentration at which 50% of the proliferation was inhibited (IC_50_) was calculated using GraphPad Prism.

### DC generation and treatment

Mouse dendritic cells (DCs) were generated by culturing mouse hematopoietic progenitors isolated from C57BL/6, TLR4^−/−^, or MyD88^−/−^ mice as previously reported [19]. Mouse DCs at 5 × 10^5^/ml in mGM-CSF-containing RPMI 1640 medium were cultured in a CO2 incubator with CT at indicated concentrations for specified time periods before harvesting culture supernatants and DCs for cytokine measurement and cytometric/signaling analysis, respectively.

### Detection of apoptosis

LLC cells in a 12-well plate (3 × 10^5^/ml/well) were cultured for 24 h with CT or NaZ_3_ (for positive control) at indicated concentrations. The cells were harvested by treating with 0.25% trypsin-2.21 mM EDTA, washed three times, and stained with an apoptosis detection kit (BMS500FI/300, eBioscience) consisting of FITC-conjugated annexin V and propidium iodide (PI) following the vendor’s recommendation. The stained samples were assayed using an LSR II (BD) flow cytometer and analyzed using FlowJo.

### Cell-cycle analysis

LLC cells with 70 ~ 80% confluency were washed and serum-starved in a CO_2_ incubator in DMEM medium containing 0.2% FBS for 48 h for synchronization. The synchronized LLC cells were plated into a 6-well plate at 5 × 10^5^/well in DMEM medium (10% FBS) containing various concentrations of CT and cultured in a CO2 incubator for 24 h. The resultant cells were washed twice with PBS and fixed in 70% ethanol for 30 min at 4 °C. After fixation, the cells were washed twice with PBS and treated with 50 µl/tube of 100 µg/ml of ribonuclease for 30 min at room temperature. Finally, 200 µl of PI at 50 µg/ml was added into each tube and the cells were analyzed using an LSR II flow cytometer.

### Treatment of LLC for signaling analysis

LLC cells were serum-starved in a CO_2_ incubator in DMEM medium containing 0.1% FBS overnight before they were treated with various concentrations of CT for 24 h. The treated cells were solubilized in 1 × SDS–PAGE sample buffer at 10^7^/ml, boiled for 5 min, and stored at − 20 °C until use.

### SDS–PAGE and western blot

Samples and pre-stained standard separated on a 4–12% NuPAGE™ (Invitrogen) were transferred onto a piece of Immobilon™ membrane (Millipore, Bedford, MA). The membranes were rinsed with TBS-T (TBS containing 0.05% Tween 20), blocked with 5% nonfat dry milk at room temperature for 1 h, and incubated with appropriately diluted (1:500 ~ 2000) 1st antibodies overnight at 4 °C. The first antibodies were rabbit IgG purchased from Cell Signaling (Beverly, MA), including anti-I-κBα (#9242), anti-GAPDH (#2118), anti-phospho-p44/42 (#9101, Thr202/Tyr204), anti-p44/42 (#9102), anti-phosphorylated p38 (#9211, Thr180/Tyr182), anti-p38 (#9212), anti-phosphorylated JNK (#9251, Thr183/Tyr185), anti-JNK (#9252), anti-phosphorylated p53 (#9284, Ser15), anti-Cdc2 (#77,055), and anti-cyclin B1 (#4138). After washing three times with TBS-T, the membranes were reacted with 1:2000 diluted HRP-conjugated goat anti-rabbit IgG (Cell Signaling, #70,741) for 1 h at room temperature, washed, and developed in SuperSignal^®^ West Dura Extended Duration Substrate (Thermo Fisher Scientific Inc., Hanover Park, IL). The images were collected using the G BOX Chemi systems (Syngene, Frederick, MD).

### Cytokine quantitation

TNFα, IL-1β, IL-10, and IL-12p70 in the culture supernatants were quantitated by human and mouse Customary Cytokine Arrays following the manufacturer’s protocol (MesoScale Diagnostics, Rockville, MD).

### LLC mouse model, treatment, and tumor tissue processing

C57BL/6 mice (female, 8 ~ 10-week-old, *n* = 5) were implanted subcutaneously with 0.2-ml sterile PBS containing 5 × 10^6^/mouse of LLC into the right flank. The appearance and growth of tumors were monitored twice a week. The greatest longitudinal diameter (length) and the greatest transverse diameter (width) of a palpable tumor were measured to the nearest 0.1 mm using a caliper. Tumor volume (mm^3^) was calculated by the formula Tumor volume = (length × width^2^)/2. LLC-bearing mice were treated every other day, starting on day 7, with intratumoral (i.t.) injection of CT alone or in combination with anti-PD-L1 at various doses for 2 weeks. In experiments determining the contribution of lymphocytes to the development of anti-LLC immune defense, LLC-bearing mice were also simultaneously treated intraperitoneal injection of 0.2 ml PBS containing 200 µg of either control rat IgG (clone 2A3, BioXcell, West Lebanon, NH), anti-mouse CD4 (clone GK1.5, BioXcell), anti-mouse CD8α (clone 53-6.72), or anti-mouse NK1.1 (clone PK136, BioXcell). In accordance with the institutional guideline, mice with big tumors (volume > 2000 mm^3^) undergoing necrosis were considered morbid and euthanized.

Tumors resected from LLC-bearing mice treated with CT alone or in combination with anti-PD-L1 were used for RNA extraction using TRIZol solution (Invitrogen). Alternatively, the tumors were dissociated into single-cell suspensions using an enzymatic cocktail consisting of collagenase I, II, and VI, deoxyribonuclease I, and elastase as previously reported [20].

### Immunostaining and flow cytometry

DCs suspended in FACS buffer (PBS containing 0.5% BSA and 0.05% NaN_3_) were blocked with 2% mouse serum on ice for 20 min and stained with various combinations of fluorophore-conjugated antibodies against human or mouse DC surface markers on ice for 30 min in the dark. Mouse DCs were stained with FITC-anti-mouse CD86 (clone GL1, TONBO Biosciences, San Diego, CA), PE-anti-mouse CD80 (clone 16-10A1, TONBO), Pacific Blue-anti-mouse CD83 (clone Michel-19, BD), and APC-anti-mouse I-A/E (clone M5/114.15.2, eBioscience). Single LLC tumor cell suspensions were stained with FITC-anti-mouse CD4 (clone GK1.5, Tonbo), PerCP-Cy5-anti-mouse-B220 (clone RA3-6B2, Tonbo), APC-anti-mouse-CD11c (clone HL3, BD), eFluor450-anti-mouse CD45 (clone 30-F11, eBioscience), and APC-Cy7-anti-mouse-CD8 (clone 53 − 6.7, Tonbo). Data of the stained samples were acquired using an LSR II flow cytometer (BD) and analyzed using the software FlowJo.

### Total RNA isolation and cDNA synthesis

RNA samples from LLC tumors were isolated by a combination use of TRIzol (Invitrogen, Cat: 1,559,026) and an RNeasy Micro Kit RNA (Qiagen, Hilden, Germany, Cat: 74,004). The purity and concentration of the extracted RNA was assessed and measured using absorption at the 260 nm wavelength with a Nanodrop ND-1000 spectrometer (Nanodrop Technologies, Wilmington, DE). Next, the cDNA was made from the isolated RNA using the RT^2^ First-Strand Kit (Qiagen, Cat: 330,401).

### Quantitative real-time polymerase chain reaction (qPCR)

The expression of target mouse genes was determined by qPCR using a LightCycler 480 II (Roche Life Sciences, Branford, CT, USA), RT^2^ SYBR Green/ROX qPCR Master Mix (Qiagen, Cat: 330,523), and the specific primer pairs for CXCL9 [Qiagen, Cat: PPM029723-200], CXCL11 [Qiagen, Cat: PPM03192C-200], Granzyme B [Qiagen, Cat: PPM05303F-200], Perforin [Qiagen, Cat: PPM34456B-200], IFNγ [Qiagen. Cat: PPM03121A-200], IL-10 [Qiagen, PPM03017C-200], and GAPDH [IDT, Cat:135,048,676]. The cycling conditions for the qPCR were: hot start for 10 min at 95 °C; amplification for 40 cycles at 95 °C for 15 s, 55 °C for 35 s, and 72 °C for 30 s; and cool down for 2 min at 37 °C. Finally, the expression levels were normalized to those of GAPDH and the data were analyzed using the ^ΔΔ^Ct method through Qiagen’s GeneGlobe Data Analysis Center.

### Statistical analysis

All experiments were performed at least three times and the results of one representative experiment or the mean of multiple experiments are shown. The difference between groups in terms of cytokine production was determined by Student’s *t* test. Differences in the in vivo tumor growth were determined by Repeated Measures of ANOVA.

## Results

### CT inhibited the proliferation of Lewis lung carcinoma (LLC) cells based on G2/M cell-cycle arrest

**Fig. 1:**
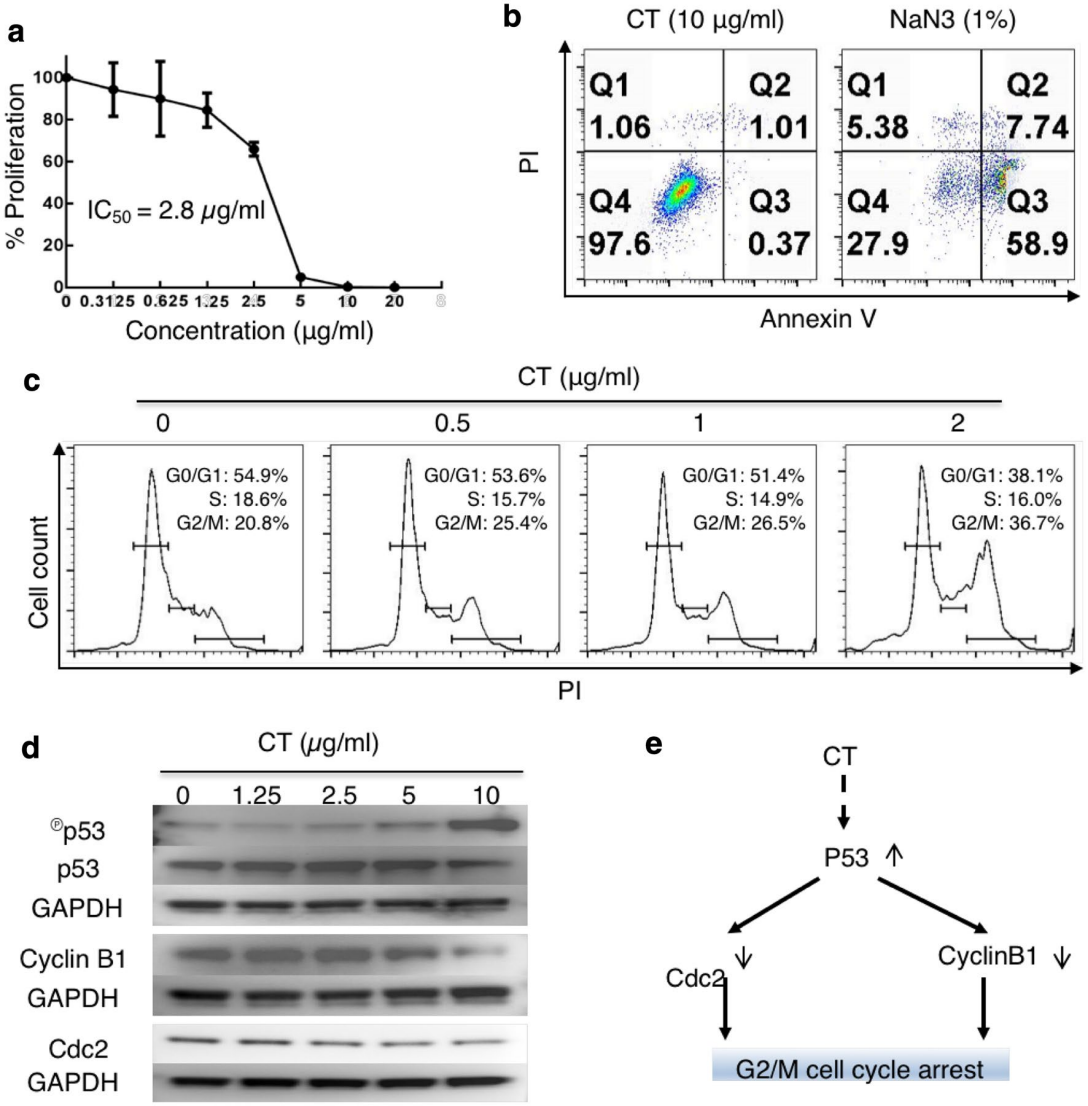
CT inhibited the proliferation of LLC through G2/M arrest with compatible intracellular signaling events. **a** LLC cancer cells were treated in triplicate in a 96-well plate for 48 h in a CO_2_ incubator with CT at indicated concentrations and their proliferation assessed by ^3^H-TdR incorporation. The % proliferation was calculated as (CPM with compound − CPM blank)/(CPM without compound − CPM blank) × 100. **b** LLC cells seeded in a 12-well plate at 3 × 10^5^/ml/well were treated with various concentrations of CT or 1% NaN_3_ (as a positive control) for 24 h in a CO_2_ incubator. Subsequently, the cells were harvested and stained with an apoptosis detection kit. Only the dot plot (PI vs annexin V) of cells treated with 10 µg/ml of CT and 1% NaN_3_ is shown. **c** Synchronized LLC treated with various concentrations of CT for 48 h in a CO_2_ incubator was stained with PI and subsequently analyzed for cell cycle. The data were graphed using FlowJo. **d** LLC cells serum-starved for 24 h were treated with indicated concentrations of CT before lysis in 1 × SDS sample buffer at 10^7^/ml. The samples were separated on a 4–12% gradient NuPAGE™ gel, transferred on a piece of Immobilon™ membrane, blocked, and reacted with anti-phospho-p53, anti-cyclin B1, or anti-Cdc2. The membrane used for probing phosphor-p53 was stripped and re-probed with anti-p53. After the images were taken, the membranes were stripped and re-probed with anti-GAPDH. **e** A chart illustrating CT-induced signaling pathways responsible for CT-induced G2/M arrest in LLC cells

Screening of a dozen of TCM compounds for their capacity to both inhibit tumor cell proliferation and promote human DC maturation identified CT. CT dose-dependently inhibited the proliferation of A549 cells, with a 50% inhibitory concentration of 0.228 µg/ml (sFig. 1a), and at 10 µg/ml, upregulated the expression of surface CD80, CD86, HLA-ABC, and HLA-DR on human monocyte-derived DCs (sFig. 1b). When LLC was tested, CT dose-dependently inhibited its proliferation, with an IC_50_ at 2.8 µg/ml (Fig. 1a). However, CT at a wide concentration range did not cause hemolysis of erythrocytes (sFig. 2) or lysis of monocyte-derived macrophages (Mφ) (sFig. 3), suggesting that CT was not cytotoxic for normal cells.

To investigate how CT inhibited LLC proliferation, LLC cells were cultured with various concentrations of CT or 1% NaN3 for 24 h and subsequently stained with FITC-conjugated annexin V and propidium iodide (PI) to detect apoptosis. NaN_3_ caused apoptotic death of LLC with a dramatic increase in the percentage of annexin V-positive cells (Fig. 1b, right plot). In contrast, even at the concentration (10 µg/ml) that completely inhibited the proliferation of LLC (Fig. 1a), CT did not induce apoptosis of LLC cells (Fig. 1b, left plot).

To determine the effect of CT on cell-cycle progression, synchronized LLC cells were treated with CT at 0 ~ 2 µg/ml for 24 h to quantitate the relative fractions of cells in every phase of the cell cycle. CT dose-dependently increased the percentage of cells in G2/M phase with concomitant reduction in the percentage of cells in G0/G1 phase, thus, demonstrating that CT treatment resulted in G2/M arrest of LLC (Fig. 1c).

LLC cells were then treated with various concentrations of CT for 24 h to identify signaling molecules that regulate cell-cycle progression. CT in a dose-dependent manner elevated the levels of phosphorylated p53, which was mirrored by a reduction of unphosphorylated p53, indicating CT activated p53 (Fig. 1d). CT also dose-dependently decreased the levels of cyclin B1 and Cdc2 (Fig. 1e). Therefore, CT induced the activation of p53 and the consequent downregulation of both Cdc2 and cyclin B1, which, in turn, prevents cell-cycle progression through the mitotic phase in LLC, resulting in G2/M arrest. CT did not alter p21 level (data not shown).

### CT-induced maturation of mouse DCs in a MyD88-dependent manner

**Fig. 2:**
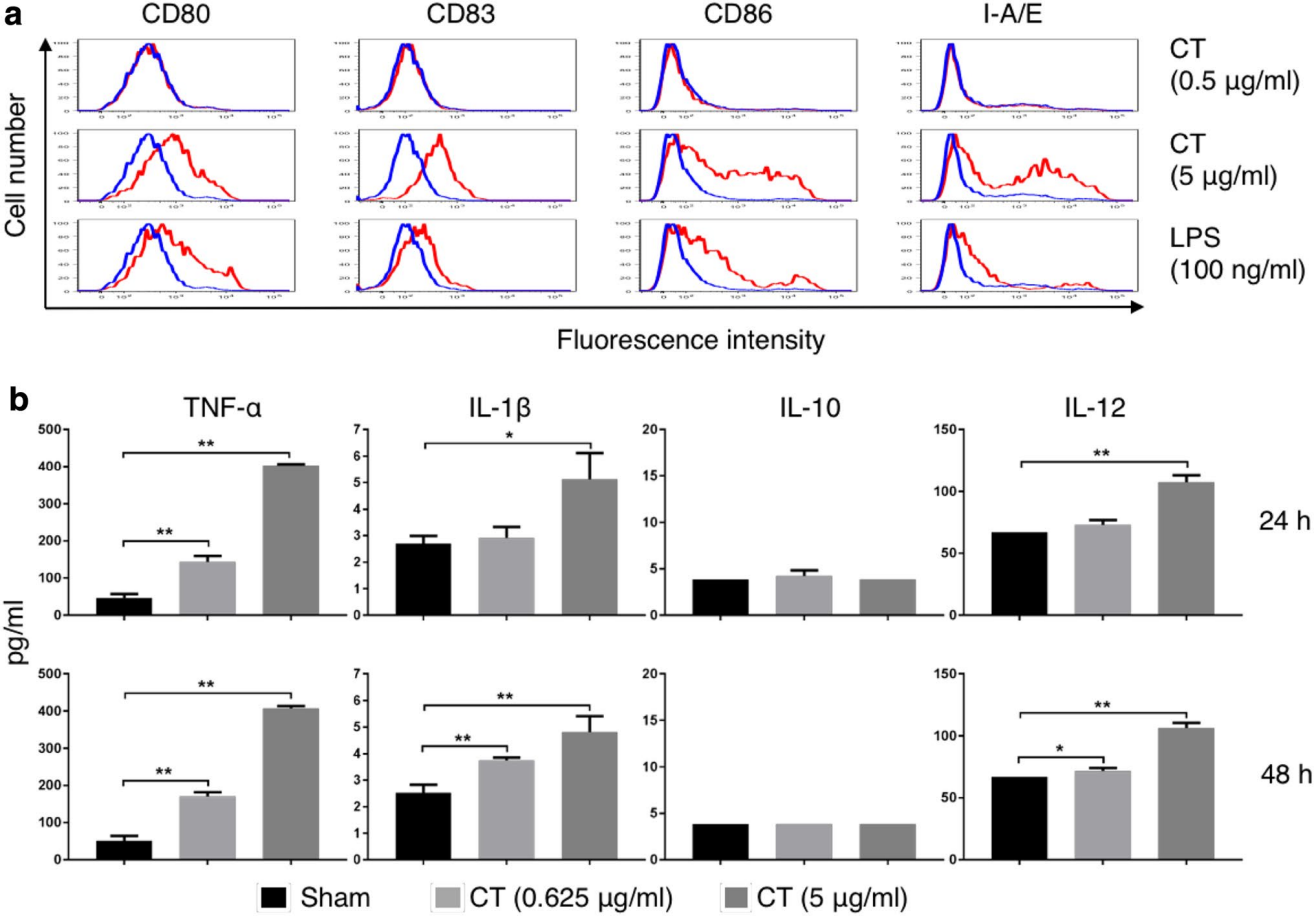
CT induced maturation of mouse DCs. **a** Mouse bone marrow-derived DCs were incubated in a CO_2_ incubator for 48 h with or without CT or LPS at the concentrations specified before they were immunostained for detection of surface marker (CD80, CD83, CD86, and I-A/E) expression by flow cytometry. Shown are the overlay histograms of sham (blue line) and treated (red line) DCs. **b** Mouse bone marrow-derived DCs were cultured in the absence (sham) or presence of various concentrations of CT for 24 or 48 h before the supernatants were harvested for the measurement of indicated cytokines. Shown is the average (mean ± SD) of three independent experiments. *p < 0.05 and **p < 0.001

The effects of CT or LPS on mouse bone marrow-derived DCs were determined. Overlay histograms showed that CT at 5 µg/ml upregulated CD80, CD83, CD86, and I-A/E, indicating that CT induced phenotypic DC maturation (Fig. 2a). Noticeably, CT at 5 µg/ml was even more effective than LPS at 100 ng/ml (Fig. 2a). In addition, CT stimulated DCs to produce TNFα, IL-1β, and IL-12p70 in a dose-dependent manner after 24 h and 48 h of treatment (Fig. 2b). Since CT did not induce IL-10 production by mouse DCs (Fig. 2b), CT-matured DCs are likely to preferentially induce Th1-polarized immune responses, which would favor the induction of antitumor immune responses.

**Fig. 3:**
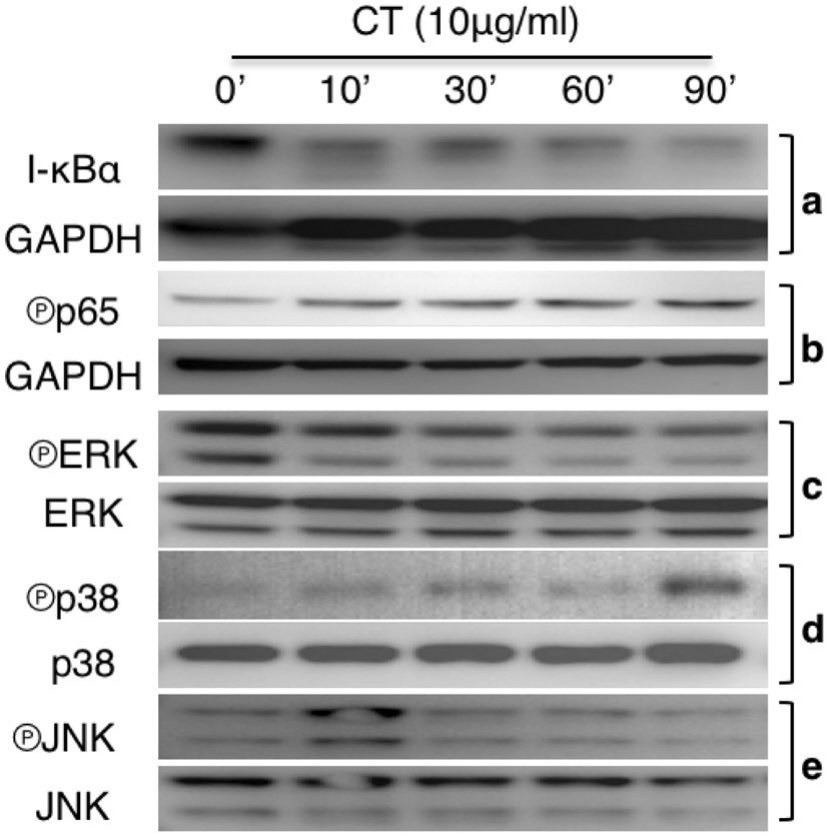
CT regulation of NF-κB and MAPK activation. **a** Mouse bone marrow-derived DCs treated with 10 µg/ml of CT for the indicated time periods were solubilized in 1 × SDS sample buffer at 10^7^/ml. The samples were separated on a 4–12% gradient NuPAGE™ gel, transferred on a piece of Immobilon™ membrane, blocked, and treated with anti-IκBα (**a**), anti-phospho-p65 (**b**), anti-phospho-Erks (**c**), anti-phospho-p38 (**d**), or anti-phospho-JNK (**e**). After the images were taken, the membranes were stripped and re-probed with anti-GAPDH, anti-Erks, anti-p38, and anti-JNK, respectively

To identify the CT-triggered signaling events, mouse bone marrow-derived DCs treated with CT at 10 µg/ml were analyzed for the activation of NF-κB and MAPKs. CT decreased I-κBα level, which became obvious within 10 min (Fig. 3a, upper panel). Re-probing the same membrane with anti-GAPDH revealed that the reduction of I-κBα was not due to uneven loading (Fig. 3a, lower panel). In addition, CT treatment also increased phosphorylation of p65 in DCs (Fig. 3b). Determination of the effect of CT on the three major MAPKs revealed that CT in a time-dependent manner lowered phosphorylated Erks (Fig. 3c). In contrast, CT induced phosphorylation of p38 and JNK with different kinetics which peaked at 90 and 10 min, respectively (Fig. 3d, e). Therefore, CT activated NF-κB, p38, and JNK, but not Erks, in mouse DCs.

**Fig. 4:**
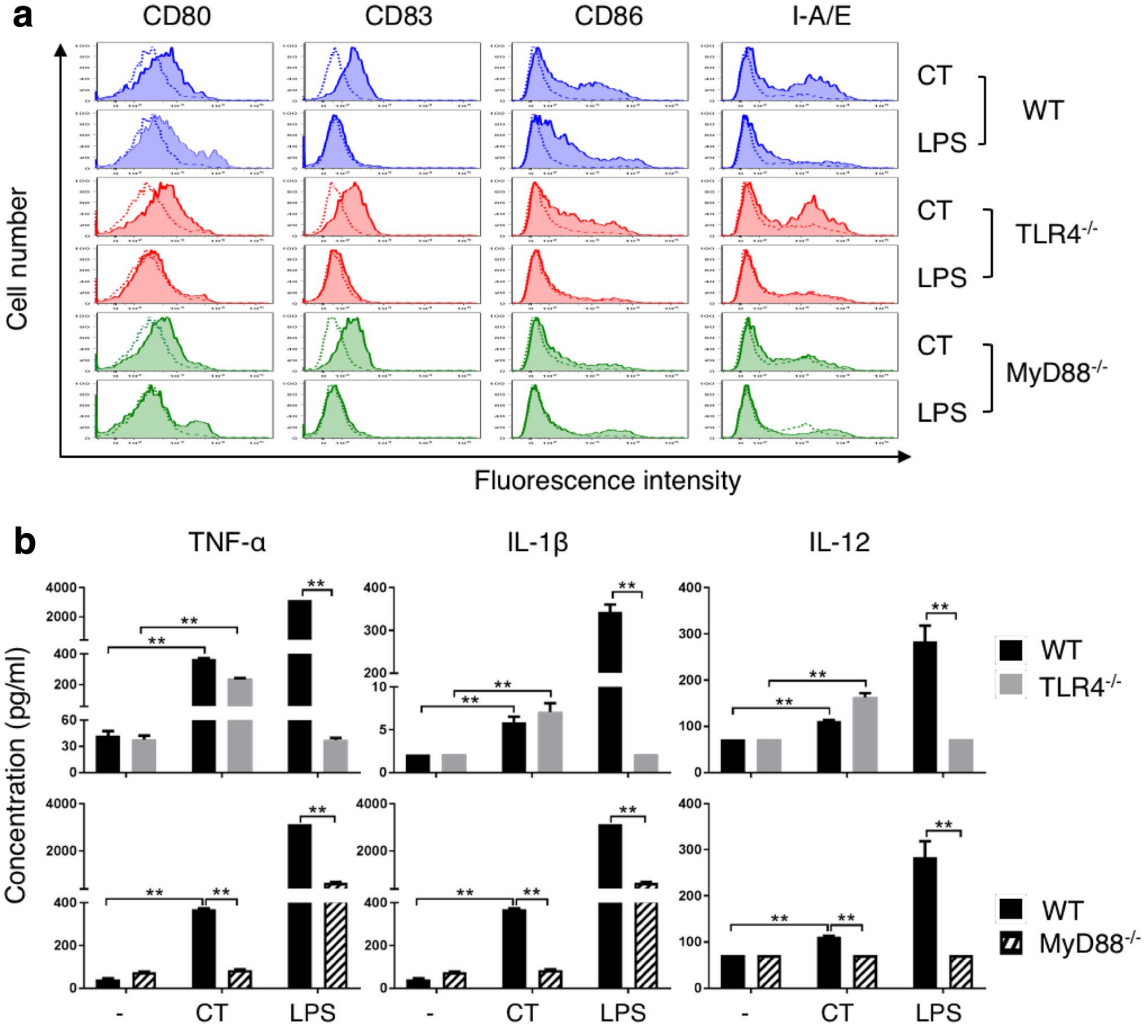
Comparison of CT-induced maturation of wild-type (WT), TLR4^−/−^, and MyD88^−/−^ mouse DCs. **a**, WT (C57BL/6), TLR4^−/−^, and MyD88^−/−^ mouse DCs were incubated in a CO_2_ incubator for 48 h with CT (5 µg/ml) or LPS (100 ng/ml) before they were immunostained for the detection of surface marker (CD80, CD83, CD86, and I-A/E) expression by flow cytometry. Shown are the overlay histograms of sham (blue line) and treated (red line) DCs. **b**, Mouse bone marrow-derived DCs were cultured in the absence (sham) or presence of CT (5 µg/ml) or LPS (100 ng/ml) for 48 h and the supernatants were harvested to assay the indicated cytokines. Shown is the average (mean ± SD) of three independent experiments. *p < 0.05 and **p < 0.001

To gain further insight into the mechanistic basis of CT-induced DC maturation, WT, TLR4^−/−^, and MyD88^−/−^ DCs were treated in parallel with CT at 5 µg/ml or LPS (100 ng/ml) for 48 h and the expression of surface markers was determined. As shown in Fig. 4a, CT induced similar upregulation of CD80, CD83, CD86, and I-A/E in WT and TLR4^−/−^ DCs, indicating that the DC-maturing effect of CT was not dependent on TLR4. These data also ruled out potential endotoxin contamination of CT used in the present study. In contrast, CT-induced upregulation of CD86 and I-A/E was markedly reduced in *MyD88*^−/−^ DCs (Fig. 4a). As anticipated, LPS-induced upregulation of CD80, CD83, CD86, and I-A/E occurred in WT DCs, but was completely deficient in TLR4^−/−^ DCs and markedly reduced in *MyD88*^−/−^ DCs (Fig. 4a). The production of proinflammatory cytokines (TNF-α, IL-1β, or IL-12p70) was absent in LPS-stimulated *TLR4*^−/−^ DCs, but there was no reduction in CT-treated TLR4^−/−^ DCs (Fig. 4b, upper panel). In contrast, *MyD88*^−/−^ DCs showed inhibition of both CT- and LPS-induced TNF-α, IL-1β, and IL-12p70 production (Fig. 4b, lower panel). These results demonstrate that CT induces DC maturation in an MyD88-dependent manner.

### Therapeutic antitumor effect of CT on LLC tumors

**Fig. 5:**
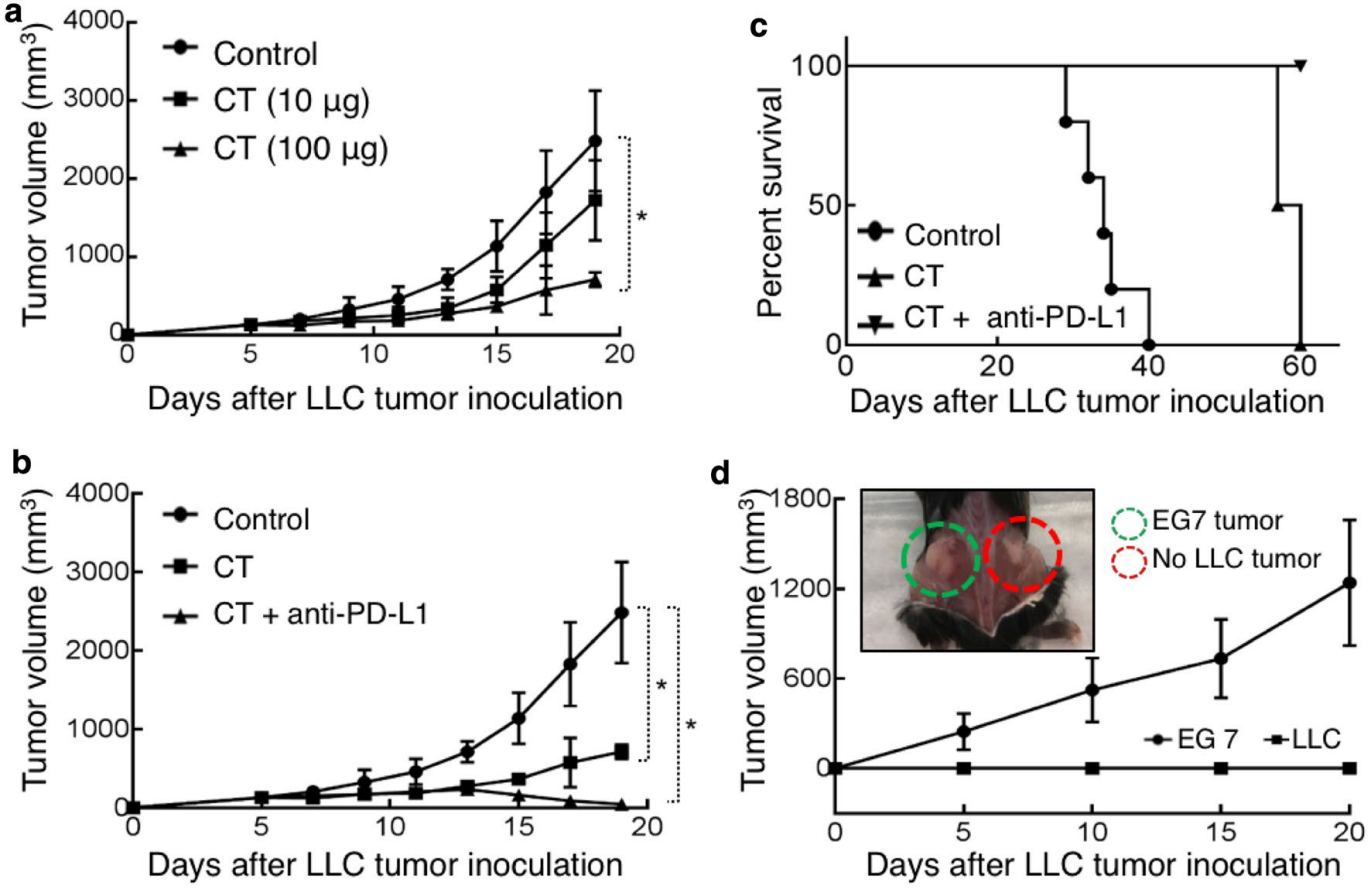
Therapeutic effect of CT on mouse LLC. **a** C57BL/6 (female, 8-week-old, *n* = 5) were subcutaneously inoculated with 5 × 10^6^/mouse of LLC into the right flank on day 1. From day 7, LLC-bearing mice were treated every other day with i.t. injection of PBS (control) or CT at the doses as indicated for 2 weeks. Tumor growth (mean ± SD) was recorded (*p < 0.05). **b, c**, LLC-bearing mice were prepared as in A and treated, starting on day 7, with i.t. injection of CT (100 µg/mouse) every other day or CT combined with i.t. injection of anti-PD-L1 antibody (10 µg/mouse) twice weekly for 2 weeks. Tumor growth (**b**) and mouse survival (**c**) were recorded. **d** The mice cured of LLC by treatment with CT + anti-PD-L1 in **c** were s.c. inoculated with 5 × 10^6^/mouse of LLC in the right flank and EG7 thymoma in the contralateral flank. The growth of tumors on both flanks was recorded. All the mice grew EG7 tumors without LLC tumors, with the photo image confirmation of one euthanized mouse at the end of the experiment (insert)

C57BL/6 mice bearing established subcutaneous LLC tumors on the flank were treated with intratumoral (i.t.) injection of CT at different doses every other day for 2 weeks and the growth of tumors was monitored. CT significantly inhibited the growth of LLC tumors at 100 µg/mouse (Fig. 5a). Lung tumor cells and tumor-infiltrating antigen-presenting cells express PD-L1, which, by interacting with PD-1 on T cells, can inactivate the effector functions of both CD4^+^ Th1 T cells and CD8^+^ CTLs, leading to the evasion of antitumor immune responses [21, 22]. To block this potential inhibitory pathway, we investigated whether a combination of CT and anti-PD-L1 antibody would exert a more robust therapeutic effect on LLC tumors. The combination of i.t. injection of CT and anti-PD-L1 at 10 µg/mouse twice weekly for 2 weeks initially arrested LLC growth, and subsequently caused LLC tumors to shrink and to be eliminated (Fig. 5b). Anti-PD-L1 at 10 µg/mouse twice weekly alone did not eliminate LLC tumors (data not shown). All LLC-bearing mice treated with i.t. PBS died by day 40, while those treated with CT survived until day 60, a significant improvement over PBS-treated group (Fig. 5c). Strikingly, all LLC-bearing mice treated with the combination of CT and anti-PD-L1 became tumor-free (Fig. 5c).

The cured tumor-free mice were subcutaneously inoculated with LLC and EG7 mouse thymoma cells on the contralateral flanks, and the formation of solid tumors on both flanks was monitored. All the mice formed solid EG7 tumors, whereas none of the mice formed LLC tumors, as illustrated by one of the mice photographed after euthanasia on day 20 (Fig. 5d). Thus, the mice cured from LLC in response to treatment with CT and anti-PD-L1 acquired long-term LLC-specific immunity.

**Fig. 6:**
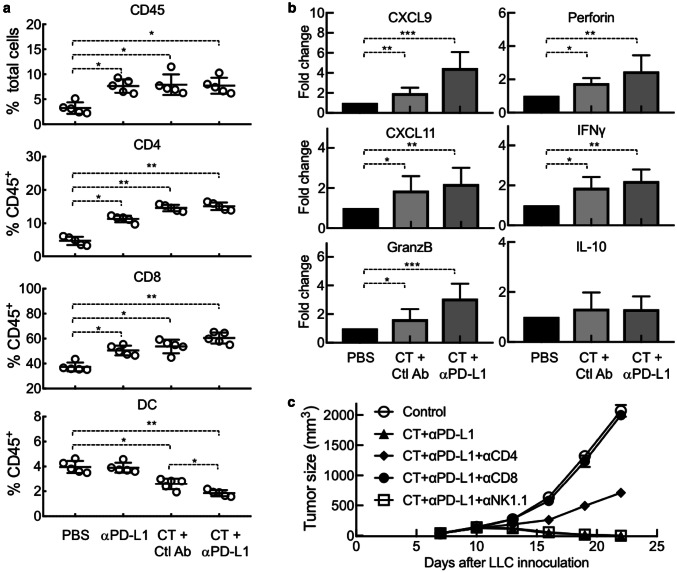
Dependence of CT’s therapeutic antitumor effect on T-cell-mediated immune defense. LLC-bearing C57BL/6 (8 weeks old, *n* = 5) mice were treated every other day with i.t. injection of CT (100 µg/mouse) and anti-PD-L1 (αPD-L1, 10 µg/mouse) alone or in combination starting on day 7. Rat IgG2b was used as the control antibody (Ctl Ab). **a** LLC tumors removed 24 h after the third treatment were dissociated into single-cell suspension, which were immunostained and analyzed for the abundance of various subsets of leukocytes. **b** RNA samples isolated from LLC tumors removed 24 h after the third treatment were quantitated for expression of indicated genes at the mRNA level by qPCR. Data in **a, b** are shown as the average (mean ± SD) of each group, with *p < 0.001 and **p < 0.0001 by *t* test. **c** LLC-bearing mice were additionally treated with i.p. injection of indicated 200 µg of leukocyte-depleting antibody once every 3 days, starting on day 5. Tumor size was monitored twice per week and tumor growth curve was plotted

LLC-bearing mice treated with CT and/or anti-PD-L1 showed a significant increase in the infiltration of CD45^+^ leukocytes, particularly CD4^+^ and CD8^+^ T cells, in their tumors (Fig. 6a). In contrast, the percentage of DCs (CD11c^+^/CD45^+^) in the tumor tissue significantly decreased in response to treatment with CT or CT plus anti-PD-L1 (Fig. 6a), suggesting that CT treatment caused in vivo DC maturation, since mature DCs migrate from tumors to draining lymph nodes [20]. qPCR analysis of LLC tumors revealed that treatment with CT significantly elevated the expression of CXCL9 and CXCL11 in LLC tumors, two chemokines responsible for the recruitment of Th1 T cells (Fig. 6b). In addition, CT treatment also elevated the expression of granzyme B, perforin, and IFNγ in the tumors (Fig. 6b), thus, demonstrating CT promotion of a Th1-polarized tumor microenvironment critical for combatting tumors. Treatment with CT in combination with anti-PD-L1 further enhanced the expression of CXCL9, CXCL11, granzyme B, perforin, and IFNγ (Fig. 6b). No significant change was detected for IL-10 expression in LLC tumors in response to treatment with CT or CT plus anti-PD-L1 (Fig. 6b).

To determine whether the therapeutic antitumor effect of CT was due to the generation of antitumor immunity, depleting antibody against mouse CD4, CD8, or NK cells was used together with treatment with CT plus anti-PD-L1. LLC tumor growth was halted by treatment with CT plus anti-PD-L1; however, simultaneous administration of anti-CD8 antibody completely abolished the therapeutic effect of CT plus anti-PD-L1 (Fig. 6c), demonstrating the critical contribution of CD8 T cells to the antitumor effect of CT. Simultaneous administration of anti-CD4 antibody also partially negated the therapeutic effect of CT plus anti-PD-L1 (Fig. 6c), suggesting that CD4 T cells also contributed. In contrast, NK cells did not seem to significantly contribute, since simultaneous administration of anti-NK1.1 antibody did not affect the therapeutic antitumor effect of CT plus anti-PD-L1 (Fig. 6c).

## Discussion

In this study, we discovered the unique capacity of CT to induce the maturation of both human and mouse DCs (sFig. 1 and Figs. 2, 4). The CT preparation used in this study contained no contaminating endotoxin, since it stimulated the maturation of both WT and TLR4 knockout DCs. Importantly, CT at concentrations capable of promoting DC production of TNFα, IL-1β, and IL-12 did not induce IL-10 production (Fig. 2). This is a remarkable feature for CT in the context of cancer treatment for two reasons. First, this suggests that DCs matured by CT preferentially promote Th1-type immune responses that are important for antitumor immune defenses. Although the induction of Th1-type immune responses is favored by DC-derived IL-12, it is inhibited by DC-derived IL-10 through the induction of Tregs [23, 24]. Second, the failure of IL-10 production by mouse DCs in tumor tissues in response to CT would also reduce the level of immunosuppression in the tumor microenvironment, since IL-10 is a potent immunosuppressive cytokine [6, 7].

The mechanism of CT-induced DC maturation involves downregulation of I-κBα and upregulation of phosphorylated p65, p38, and JNK in DCs (Fig. 3). Reduction of I-κBα level and phosphorylation of p65 allow p50/p65 complex to translocate from the cytosol to the nucleus to interact with promoters with NF-κB-binding sites, which, in turn, promotes the transcription of many cytokine genes such as TNFα, IL-1β, and IL-12 [25, 26]. Activation of p38 and JNK in DCs is critical for the upregulation of the expression of surface costimulatory and MHC molecules as well as production of IL-12 [23, 25]. Therefore, both NF-κB and MAPK signaling pathways are involved in CT-induced DC maturation. The signaling pathway upstream of NF-κB and MAPK requires MyD88, because CT-induced DC upregulation of CD86 and I-A/E as well as induction of TNFα, IL-1β, and IL-12 were greatly compromised in MyD88^−/−^ DCs (Fig. 4). How CT stimulates a MyD88-dependent signaling pathway that results in DC maturation is under current investigation. Since MyD88 is the adaptor protein that acts downstream of many Toll-like receptors and the IL-1 receptor superfamily, it is speculated that CT may use one of the receptors.

The anti-proliferative effect on both human (sFig. 1) and mouse (Fig. 1) lung cancer cells was not based on an overall cytotoxic effect, because CT at concentrations as high as 20 µg/ml did not lyse human erythrocytes and was not cytotoxic for human macrophages (sFigs. 2 & 3). Inhibition of LLC proliferation was based on induction of cell-cycle arrest at the G2/M phase (Fig. 1). Measurement of cell-cycle regulators revealed that CT dramatically activated p53 in LLC cells (Fig. 1d). p53 is an important tumor suppressor that regulates both G1 and G2/M cell-cycle checkpoints of mammalian cells [27, 28]. p53 induces G1 arrest by promoting the transcription and expression of p21^WAF1/CIP1^, a cyclin-dependent kinase (Cdk) inhibitor [27, 29]. Binding of p21^WAF1/CIP1^ to a number of cyclin/Cdk complexes inhibits their kinase activities, resulting in hypophosphorylation of Rb, sequestration of E2F, and failure to activate E2F-responsive genes leading to G1 arrest [28, 30]. Consistent with the fact that CT did not induce G1 arrest in LLC cells (Fig. 1d), CT treatment of LLC cells did not alter the cellular levels of p21^WAF1/CIP1^ or the phosphorylation of Rb (data not shown). p53 transcriptionally represses cyclin B1 and inhibits Cdc2, and because the cyclin B1/Cdc2 complex is required for cell-cycle entry into mitosis, this results in G2/M arrest [27, 28, 31]. In full agreement with CT-induced G2/M arrest, CT-induced activation of p53 was also accompanied by reduction of cyclin B1 and Cdc2 (Fig. 1d). Thus, the signaling pathway responsible for CT-induced G2/M arrest in LLC cells involves activation of p53 that downregulates cyclin B1 and Cdc2, leading to G2/M cell-cycle arrest (Fig. 1e). It was previously reported that treatment of human lung cancer cell line A549 with CT also resulted in downregulation of cyclin B1 and G2/M cell-cycle arrest [32]. Therefore, it is likely that induction of G2/M cell-cycle arrest is a common pathway for CT-induced inhibition of proliferation of both human and mouse lung cancer cells. It remains to be determined how CT causes phosphorylation and activation of p53 in LLC lung cancer cells.

In addition to inducing G2/M arrest, CT treatment also caused apoptotic cell death of human lung cancer A549 cells (data not shown), confirming a previous report [32]. Very recently, CT has also been reported to inhibit the proliferation of A549 human lung cancer cells through a signaling pathway involving induction of reactive oxygen species, activation of JNK, downregulation of mTOR, and ultimately formation of pro-death autophagy [33]. Irrespective of the underlying mechanistic basis, administration of CT into nude mice harboring human lung cancers inhibited the growth of xenograft tumors [32, 33], indicating that CT exhibits direct anti-proliferative effect against lung cancer cells in vivo.

Given the unique dual effects of CT on lung cancer cells and DCs, we hypothesized that CT would exhibit immunotherapeutic anti-lung cancer effect in immunocompetent mice by augmenting the generation of antitumor immunity. Indeed, CT exhibited a remarkable therapeutic effect on established LLC tumors when used alone and more so in combination with anti-PD-L1 antibody in mice (Fig. 5). It is very encouraging that mice bearing established s.c. LLC tumors were cured by a combination of CT and low doses of anti-PD-L1 (Fig. 5). The data showing that the resultant tumor-free mice were resistant to re-challenge with LLC, but not B16 melanoma, demonstrate that treatment with CT plus low doses of anti-PD-L1 promoted the generation of LLC-specific antitumor immune responses and immunological memory.

How does the combination of CT and anti-PD-L1 eradicates LLC tumors? CT administered into LLC tumors would likely promote LLC-specific immune responses by stimulating the maturation of LLC-infiltrating DCs, followed by DC trafficking from tumor tissue to the draining lymph nodes, and the induction of generation of LLC-specific effector T cells. Once generated in the draining lymph nodes, LLC-specific effector CD4 (Th1) and CD8 (CTLs) would migrate into tumor tissue to produce IFNγ and kill tumor cells. In agreement with this scenario, CT treatment of LLC tumors resulted in maturation of DC and significantly lowered the abundance of DCs in the tumors (Fig. 6a), presumably due to the migration of mature DCs to draining lymph nodes [20]. In addition, LLC tumors treated with CT showed elevated infiltration of T cells (Fig. 6a) and a profile of gene expression typical of Th1 polarization (Fig. 6b). Furthermore, the therapeutic anti-LLC effect of CT in combination with anti-PD-L1 was remarkably reduced by depleting CD4 or CD8 T cells (Fig. 6c). In addition to promoting tumor killing, IFNγ also upregulates PD-L1 expression on tumor cells, which, in turn, inhibits the effector function of CTLs by interacting with PD1 on CTLs [34, 35]. Administration of anti-PD-L1 could blockade PD-L1-PD1 interaction to prevent silencing of CTLs, allowing sustained killing of LLC tumor cells. Thus, combination of CT and anti-PD-L1 promotes more robust Th1 polarized environment in the tumors (Fig. 6b) and manifests more potent antitumor effects than CT alone, resulting in complete elimination of LLC tumors (Figs. 5, 6c). Since CT is also capable of inhibiting the proliferation of human A549 lung cancer cells (sFig. 1, 32, 33 and Refs. [32, 33]) and inducing the maturation of human DCs (sFig. 1), CT may be similarly effective against human lung cancers. Therefore, CT alone or in combination with anti-PD-L1 may provide a new treatment for human lung cancers.

CT has previously been reported to inhibit the proliferation of diverse types of tumor cells in vitro, such as prostate cancer [36–38], human rhabdomyosarcoma [39], human leukemia [40], breast cancer [41], pancreatic cancer [42], and colon cancer [43]. A very recent paper even reported that CT treatment of prostate cancer stem cells could inhibit their proliferation and tumorigenesis by downregulating the expression of stemness genes including Nanog, SOX2, Oct4, and CXCR4 [44]. We have confirmed that CT can inhibit the proliferation of multiple human and mouse cancer cell lines (data not shown). Although it remains to be established, it is likely that CT may promote antitumor immunity to other types of cancers in addition to lung cancer. More recent studies reveal that CT is also effective against mouse Hepa1-6 hepatoma as described in a parallel paper by Han et al. entitled “Inhibition of murine hepatoma tumor growth by cryptotanshinone involves TLR7-dependent activation of macrophages” (Cancer Immunol Immunother, 2019).

In conclusion, we have identified a TCM-derived compound CT with unique dual capabilities of inhibiting the proliferation of lung cancer cells and inducing DC maturation. CT is effective for the treatment of established LLC cancers alone or even more effectively in combination with anti-PD-L1 in immunocompetent mice. Since the cured mice exhibit specific immunity to the treated tumors, this represents the first report that the enhancement of antitumor immunity by CT is a crucial contributor to its therapeutic efficacy.

## Cell line authentication

All cell lines used in the present study were obtained from the American Type Culture Collection (Manassas, VA). The cell lines were expanded and cryopreserved according to the culture and cryopreserving conditions recommended by American Type Culture Collection. Lewis lung carcinoma (LLC) cell line was passaged and maintained in DMEM medium (Meditech, Manassas, VA) supplemented with 10% FBS (Hyclone, Logan, UT), 2 mM L-glutamine, 25 mM HEPES, 100 U/ml penicillin, and 100 µg/ml streptomycin. A549, a human lung carcinoma cell line, and EG7, a cell line derived from EL4 thymoma, were passaged and maintained in RPMI 1640 medium (Meditech) supplemented with 10% FBS, 2 mM glutamine, 25 mM HEPES, 1.5 g/l sodium bicarbonate, 4.5 g/l glucose, 1 mM sodium pyruvate, 100 U/ml penicillin, 100 µg/ml streptomycin, and 50 µM 2-ME. For any experiment involving the use of a cell line, one of the cryopreserved vials was freshly thawed and passaged for three times before the cells were used, and, therefore, cell line authentication was not necessary.

### Electronic supplementary material

Below is the link to the electronic supplementary material.

Supplementary material 1 (PDF 594 KB)

## Abbreviations

CT: Cryptotanshinone
Cdk: Cyclin-dependent protein kinase
Cdc2: Cyclin-dependent protein kinase 2
CTL: Cytotoxic T lymphocyte
Erk: Extracellular signal-regulated kinase
JNK: c-Jun N-terminal kinase
LLC: Lewis lung carcinoma
Mϕ: Macrophage
MAPK: Mitogen-activated protein kinase
PD1/PD-L1: Programmed cell death protein 1/programmed death ligand 1
PI: Propidium iodide
TCM: Traditional Chinese medicine
WT: Wild-type
