# Supplementary material for: Cryptotanshinone has curative dual anti-proliferative and immunotherapeutic effects on mouse Lewis lung carcinoma
Source: Cancer Immunol Immunother. 2019 Apr 10;68(7):1059–71. doi: 10.1007/s00262-019-02326-8 (PMC6584267; doi:10.1007/s00262-019-02326-8)
Supplement: Supplementary file 1 — Supplementary material 1 (PDF 594 KB) [file 262_2019_2326_MOESM1_ESM.pdf]

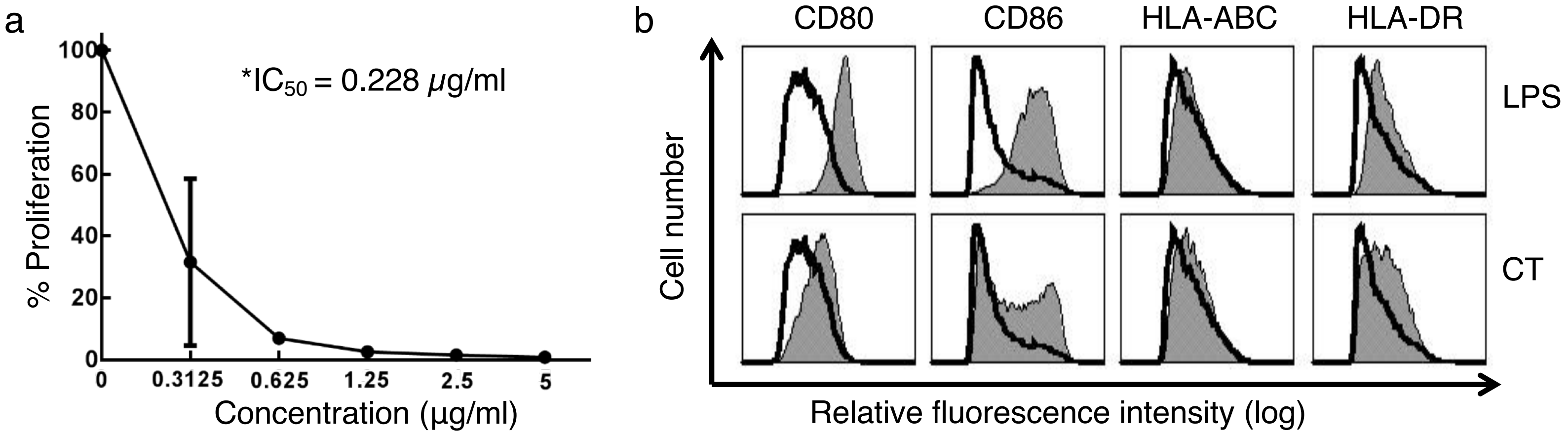

sFig. 1. CT exhibited the dual capacities of inhibiting the proliferation of human lung cancer cells and inducing the maturation of human DCs. **a,** A549 human lung cancer cells seeded in a 96-well plate at 4×10^3/well were treated in triplicate with indicated concentrations of CT for 48 h in a CO_2 incubator (37°C humidified air containing 5% CO_2) and pulsed with 0.5 µCi/well of ^{3}H-TdR for the last 4 h. After cell harvest and β scintillation counting, the % proliferation was calculated as % proliferation = (CPM with compound - CPM blank) ÷ (CPM without compound - CPM blank) × 100. *IC_{50} was the calculated concentration at which 50% of the proliferation was inhibited. **b,** Human DCs were cultured in a CO_2 incubator for 48 h in the absence (open area) or presence (grey area) of LPS at 100 ng/ml or CT at 10 µg/ml before they were immunostained and analyzed by flow cytometry. Shown are the overlay histograms illustrating the expression of surface ostimulatory (CD80, and CD86) and MHC (HLA-ABC and HLA-DR) of sham (solid line) and treated (grey area) DCs.

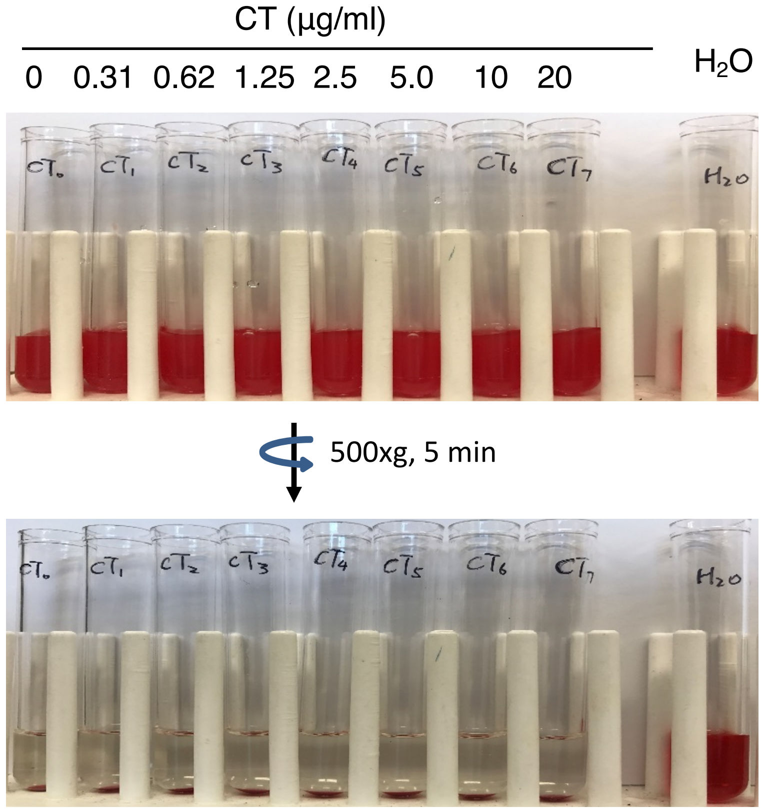

sFig. 2. CT did not cause hemolysis. Human erythrocytes were suspended in PBS at 2% (vol./vol.) containing CT at various concentrations or H_2O (positive hemolysis control). All the tubes were incubated at room temperature for 30 min, and then centrifuged at 500x*g* for 5 min. The photo images before and after centrifugation were recorded. In the tubes treated without or with CT (0.31-20 µg/ml), the erythrocytes sedimented to the bottom of the tubes and the supernatant remained clear, demonstrating the lack of hemolysis.

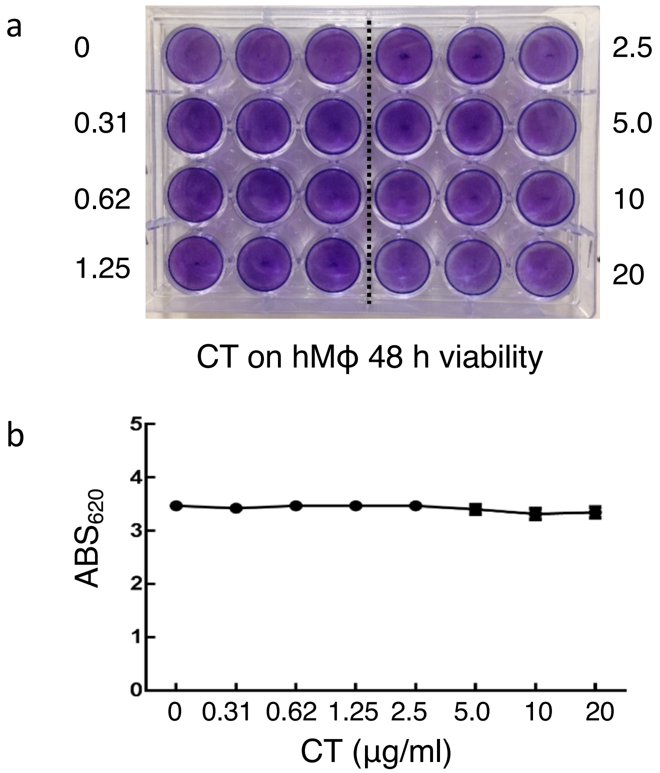

sFig. 3. CT did not cause lysis of human macrophages (hMϕ). Purified monocytes were cultured in a CO_2 incubator in RPMI 1640 supplemented with 10% FBS, 2 mM glutamine, 25 mM HEPES, 100 U/ml penicillin, 100 mg/ml streptomycin, 50 mM 2-mercaptoethanol and 50 ng/ml rhM-CSF (PeproTech) in a 48-well tissue culture plate at 2 x 10^5/well for 7 days with 50% medium replacement on day 3 and day 5. Subsequently, CT was added into triplicate wells at concentrations as specified and incubated for another 48 h. The plate was stained with 1% Toluidine blue (Sigma, St. Louis, MO) dissolved in 1% sodium tetraborate (Sigma) for 45 min at room temperature, followed by washing with distilled water. After air-drying, the plate was photo-imaged (a). The dye in the plate was solubilized by adding 0.5 ml of 1% SDS and the absorbance at 620 nm of each well was measured using a spectrometer. The results are shown as the average of ABS260 of triplicate wells (b). Almost identical ABS620 readings for all groups indicated that CT was not toxic for hMϕ.
